# Lactic Acid Bacteria in Wine: Technological Advances and Evaluation of Their Functional Role

**DOI:** 10.3389/fmicb.2020.612118

**Published:** 2021-01-15

**Authors:** Carla Virdis, Krista Sumby, Eveline Bartowsky, Vladimir Jiranek

**Affiliations:** ^1^Department of Wine Science, University of Adelaide, Urrbrae, SA, Australia; ^2^Australian Research Council Training Centre for Innovative Wine Production, Urrbrae, SA, Australia; ^3^Lallemand Australia, Edwardstown, SA, Australia

**Keywords:** MLF, LAB, wine aroma, quality, enzymes, management

## Abstract

Currently, the main role of Lactic Acid Bacteria (LAB) in wine is to conduct the malolactic fermentation (MLF). This process can increase wine aroma and mouthfeel, improve microbial stability and reduce the acidity of wine. A growing number of studies support the appreciation that LAB can also significantly, positively and negatively, contribute to the sensorial profile of wine through many different enzymatic pathways. This is achieved either through the synthesis of compounds such as diacetyl and esters or by liberating bound aroma compounds such as glycoside-bound primary aromas and volatile thiols which are odorless in their bound form. LAB can also liberate hydroxycinnamic acids from their tartaric esters and have the potential to break down anthocyanin glucosides, thus impacting wine color. LAB can also produce enzymes with the potential to help in the winemaking process and contribute to stabilizing the final product. For example, LAB exhibit peptidolytic and proteolytic activity that could break down the proteins causing wine haze, potentially reducing the need for bentonite addition. Other potential contributions include pectinolytic activity, which could aid juice clarification and the ability to break down acetaldehyde, even when bound to SO_2_, reducing the need for SO_2_ additions during winemaking. Considering all these findings, this review summarizes the novel enzymatic activities of LAB that positively or negatively affect the quality of wine. Inoculation strategies, LAB improvement strategies, their potential to be used as targeted additions, and technological advances involving their use in wine are highlighted along with suggestions for future research.

## Introduction

The fruit of the grapevine, *Vitis vinifera*, was first transformed into wine sometime between 8500BC and 4000BC (Varriano, 2010). However, wine, as we know it nowadays, is a complex beverage in which many key elements shape the final product. These key elements include the quality of the grapes and their varietal and clonal genotype, the yeasts and bacteria conducting the alcoholic fermentation (AF) and the malolactic fermentation (MLF), respectively, the aging vessels and the winemaking techniques (Styger et al., 2011).

The main role of lactic acid bacteria (LAB) in wine has traditionally been to perform the conversion of malic acid to lactic acid. In the last decades, various papers have shown that LAB metabolism also involves a large array of secondary enzymatic activities capable of generating many volatile secondary compounds (Matthews et al., 2004; Sumby et al., 2010; Bartowsky and Borneman, 2011; Michlmayr et al., 2012; Cappello et al., 2017; Takase et al., 2018).

Although it is evident that wines originating from a specific grape variety display particular characters that distinguish them from other varieties, in many cases these active-flavor compounds are not detectable at pre-fermentative stages (Swiegers et al., 2005). Often, they are instead the product of the metabolism of yeast and bacteria and are modified and released in wine during the fermentation processes. Enzymes from LAB that can exert their activity in wine include glycosidases, esterases, proteases and others (Liu, 2002). The activity of these enzymes can significantly add to the appearance, flavor, texture and aroma of wine, ultimately, helping to define its structure (Swiegers et al., 2005).

**FIGURE 1:**
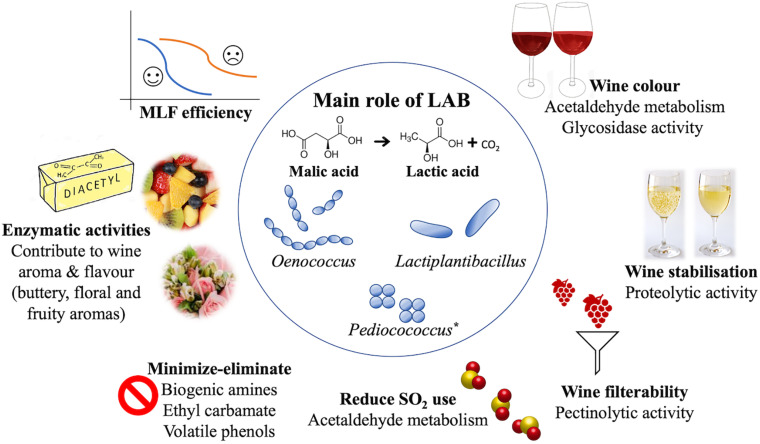
The present and potential future for LAB in winemaking, as discussed in this review. MLF (central) is the main role of LAB. However, there are many other features to consider (outer circle), with the utilization of selected starter cultures, as displayed above. *Given the ability to perform MLF, *Pediococcus* has been acknowledged in this figure. However, due to the variability of its contribution to wine, *Pediococcus* is not currently used as a starter culture and may be tentatively suggested as MLF starter for the future.

In this review we examine the current literature with regards to the functional role of LAB in winemaking (Figure 1) including; inoculation strategies, modification of wine aroma, impact on wine color, potential novel uses to aid winemaking processes, and their overall effect on the wholesomeness of wine. Finally, we discuss current limitations and future prospects.

## The Role of Lactic Acid Bacteria in Winemaking

### The Malolactic Fermentation

Once the primary fermentation has finished, or simultaneously in some cases, most red, some white and sparkling wines undergo a secondary microbial fermentation. During MLF, LAB convert the dicarboxylic malic acid into the monocarboxylic lactic acid and carbon dioxide (Figure 1) with no free intermediary products (Ribéreau-Gayon et al., 2006a). MLF typically achieves a more palatable wine by reducing the tart taste of malic acid. Additionally, MLF reduces the amount of residual nutrients able to support microbial growth making the wine more stable prior to being bottled (Lonvaud-Funel, 1999).

Several factors affect the performance of LAB in wine, their growth, their ability to complete MLF and their metabolic properties (Sumby et al., 2019). Some known limiting factors are high ethanol content, low pH, sub-optimal temperatures and high SO_2_, however, their inhibiting effect can be potentiated by their synergistic action (Lerm et al., 2010).

### Inoculation Strategies

Malolactic fermentation can be facilitated by autochthonous LAB or can be induced by inoculating the wine with selected bacterial starters (Table 1). Before the use of MLF starters became a common winemaking practice, a secondary fermentation in wine was often enabled by the microbial populations that originated in the vineyard or winery and were brought to the wine via the grape skin or the winery vessels (Bokulich et al., 2016). Although not common, uninoculated MLF performed by autochthonous LAB is still used in winemaking (Table 1), mainly with the aim of producing wines with a regional character or a particular style (Bokulich et al., 2016). However, when MLF is uninoculated and carried out by autochthonous bacteria, the conversion of malic acid into lactic acid can be slow or incomplete, or undesired volatile compounds and potentially hazardous compounds can be produced (Lonvaud-Funel, 1999). The use of bacterial starters can help minimize these risks (Lonvaud-Funel, 1999). Considerable research has been dedicated to identifying robust starters with desirable metabolic activity (Lerm et al., 2011). More recently, efforts have been directed to exploring the biodiversity of wine associated geographic areas, with the aim of finding new LAB starters with a high degree of adaptation to the specific niches (Miranda-Castilleja et al., 2016; López-Seijas et al., 2020). For example, two potential new autochthonous MLF starters with interesting β-glucosidase activity, *Lacticaseibacillus paracasei* (formerly known as *Lactobacillus paracasei*) UVI-2 and *Lentilactobacillus hilgardii* (formerly known as *L. hilgardii*) UVI-23, have been identified from Albariño grapes in Val do Salnés, Spain (López-Seijas et al., 2020). In another study, the isolation of numerous *Lactiplantibacillus plantarum* (formerly known as *Lactobacillus plantarum)* highlighted a great intra-species genetic variability in North Patagonia, Argentina, and suggested also their potential use as MLF starter cultures for regional wines (Bravo-Ferrada et al., 2013). This is especially interesting considering that the regional identity of wines, or terroir, can be an important factor in increasing the value of the final product (Bartowsky et al., 2015).

**TABLE 1:** Overview of the main LAB of enological interest, and their main roles in wine; past, present, and future.

	**Strains used**	**When**	**Major roles**	**Other roles**	**References**
Past	• Autochthonous LAB	Not controllable	Softer and more aromatic wine		Bokulich et al., 2016

Present	• Single bacterial starter cultures: *O*. *oeni* *L. plantarum* • Mixed bacterial starter cultures	During and after AF	Softer mouthfeel, Higher microbiological stability Lower acidity	Production of diacetyl, esters, aromatic thiols and other aromatic compounds, Liberation of glycoside-bound aromatic compounds	Bartowsky and Borneman, 2011; Krieger-Weber et al., 2020
	• Autochthonous LAB	Not controllable	Softer and more aromatic wine	Development of regional character	Bokulich et al., 2016

Future	• Single bacterial starter cultures: *O*. *oeni* *L. plantarum* *Pediococcus spp.* Other LAB? • Mixed bacterial starter cultures • Autochthonous, LAB starters	Before, during and after AF	Softer mouthfeel, Higher microbiological stability, Lower acidity, Production of diacetyl, esters, aromatic thiols and other aromatic compounds, Liberation of glycoside-bound aromatic compounds	Reduction of Acetaldehyde content in wine, Reduction of biogenic amine content in wine, Protein stability, Juice clarification, Reduction of SO_2_ required in winemaking, More properties?	Wade et al., 2018; Iorizzo et al., 2016; Wells and Osborne, 2011; López-Seijas et al., 2020

When selected bacterial starters are used (Table 1), the winemaker can opt for a sequential inoculation with yeast, where *Oenococcus oeni* is inoculated at the end of AF, or for a coinoculation strategy, where LAB starters are inoculated simultaneously or shortly (24–48 h) after the beginning of AF (Lucio et al., 2017; Bartle et al., 2019; Sumby et al., 2019). The main advantages of the coinoculation technique, compared to the more traditional sequential inoculation, are the potentially positive contribution to wine composition and the reduction of microbial spoilage risk (Sumby et al., 2019). Reducing the overall AF and MLF time allows the winemaker to protect the wine with SO_2_ additions earlier in the winemaking process, thus avoiding the production of volatile phenols from spoilage microorganisms such a*s Brettanomyces bruxellensis* (Sumby et al., 2019). During coinoculation, the interactions between yeasts and bacteria may affect the efficiency of MLF and the sensory properties of the final wine (Bartle et al., 2019; Du Plessis et al., 2019). For more information regarding yeast and bacteria interactions, refer to the recent review from Bartle et al. (2019). Lastly, bacteria can be inoculated before yeast. Although not common this technique has proven to be very efficient in promoting MLF, especially when *Liquorilactobacillus mali* (formerly known as *Lactobacillus mali*), *L. paracasei, Liquorilactobacillus satsumensis* (formerly known as *Lactobacillus satsumensis*) *and L. plantarum* strains were used as starters (Lucio et al., 2017). Amongst the above mentioned genera, the best results in terms of growth in juice and malic consumption efficiency were obtained with *L. plantarum* (Lucio et al., 2017).

In recent years, mixed inoculation strategies have also been trialed (Table 1). The use of blended cultures of *L. plantarum* and *O. oeni* as MLF starters, can facilitate a rapid consumption of malic acid, whilst contributing significantly to the volatile profile of wine (Brizuela et al., 2018). Preparations comprising mixtures of *L. plantarum* and *O. oeni*, recommended for coinoculation use, are commercially available (for example Anchor Oenology).

The timing of inoculation leads to different aroma compounds being released in wine, qualitatively and quantitatively modifying wine profiles (Lasik-Kurdyś et al., 2018). However, independently from the inoculation time, the key of the success of MLF seems to be the correct management of the inoculation technique (Lombardi et al., 2020). For example, pre-adapting the bacterial starters to sub-optimal pH (5.0) can improve the consumption of malic acid (Lombardi et al., 2020).

### Lactic Acid Bacteria Used in Winemaking

Lactic Acid Bacteria are Gram-positive bacteria, grouped in the phylum Firmicutes, class Bacilli, order Lactobacillales (Holzapfel et al., 2014). Several genera of the family *Lactobacillaceae* are used in the food industry and are involved in the production of numerous fermented foods, such as yogurt, cheese and sauerkraut (Szutowska, 2020). *Oenococcus, Leuconostoc*, and *Pediococcus*, within the formerly known Leuconostocaceae family, and the formerly known genus *Lactobacillus*, within the formerly known Lactobacillaceae family, are the only genera associated to wine (Holzapfel et al., 2014; Zheng et al., 2020).

Recently the description of LAB has been amended, following a modern multifactorial approach used to re-evaluate the taxonomy of these microorganisms (Zheng et al., 2020). Core genome phylogeny, physiological and metabolic criteria, and the ecology of the organisms are some of the parameters used for this evaluation (Zheng et al., 2020). The formerly known genus *Lactobacillus* has been restructured into 25 new genera and the former *Lactobacillaceae* and *Leuconostocaceae* families have been fused into a new larger *Lactobacillaceae* family (Zheng et al., 2020).

Lactic acid bacteria can be both detrimental and beneficial to the quality of wine (Bartowsky, 2009). Their performance in wine is related to the specific species and strain genetics but also to many other factors including environmental conditions and microbial interactions (Cappello et al., 2017; Devi and Ka, 2019). At present, *O. oeni* is one of the three, and the most known, species in the *Oenococcus* genus (Lorentzen and Lucas, 2019). Due to its high tolerance for low pH, high ethanol concentrations and scarcity of nutrients, *O. oeni* is the main LAB of choice in winemaking (Bartowsky, 2005). However, with increasing temperatures during growth and harvest, and a consequent rising pH trend for many wines, other LAB have the potential to become a valid alternative to *Oenococcus*, playing an important role in the modifications of wine aroma (Du Toit et al., 2010; Mira and de Orduña, 2010; Berbegal et al., 2019; Krieger-Weber et al., 2020; López-Seijas et al., 2020; Shao-Yang et al., 2020; Sun et al., 2020). Above all, *Lactiplantibacillus* strains, with their fast consumption of malic acid (up to 3 g/L in 2–4 days) and the suppression of the activity of other spontaneous LAB populations, are an ideal starter choice for the winemaker (Du Toit et al., 2010; Krieger-Weber et al., 2020). Furthermore, as *L. plantarum* is homofermentative for hexoses it does not produce volatile acidity (VA) through sugar metabolism. Currently, only a few freeze-dried starter cultures of *L. plantarum* are commercially available and their use is especially recommended for coinoculation strategies in wines with high pH (>3.4) and a high risk of autochthonous LAB contamination^1,^^2^.

Lastly, the genus *Pediococcus* is generally considered a spoilage microorganism in wine (Wade et al., 2018). *P. damnosus, P. inopinatus, P. parvulus*, and *P. pentosaceus* have been reported to produce excessive diacetyl, exopolysaccharides, biogenic amines, acrolein and more generally off-odors, flavors and textures, thus contributing detrimentally to wine quality (Wade et al., 2018). However, recent findings have shown that the presence of *Pediococcus* species in wine does not always lead to spoilage and that some species and strains within this genus may contribute positively to wine aroma and can inhibit the formation of 4-ethylphenol from the spoilage yeast, *B. bruxellensis* (Strickland et al., 2016; Wade et al., 2018). Given the variability of the contribution to wine from *Pediococcus* strains, further studies on this genus are of crucial importance. Understanding the differences between strains, and the interactions of this genus with other microorganisms, could open up the possibility of using selected *Pediococcus* starter cultures, potentially reconsidering its role in winemaking.

## How Can Lab Improve Wine Aroma?

### Citrate Metabolism

Oenologically, one of the major aroma compounds associated with LAB is diacetyl, which originates from citrate fermentation (Bartowsky and Borneman, 2011). At low concentrations (1–4 mg/L) diacetyl confers the typical buttery character to wine, while at high concentrations (>5–7 g/L) it is associated with undesirable aromas (Bartowsky and Henschke, 2004). A 2002 survey showed that the concentration of diacetyl in wine varies widely, ranging from 0.3 to 0.6 mg/L in Chardonnay wines and from 0.3 to 2.5 mg/L in red wines (Bartowsky et al., 2002). The sensory threshold can vary greatly across different types of wines and is greatly affected by the presence in wine of other compounds such as sulfur dioxide (Bartowsky et al., 2002).

Referring to their ability to degrade citrate, LAB can be classified in cit^+^ and cit^–^. Cit^+^ strains have all the genes that encode for the necessary permease and the lyase subunits needed to degrade citrate, leading to the production of pyruvate. Conversely, cit^–^ strains may lack one or more genes in this metabolic pathway and cannot degrade citrate, however, they can still produce diacetyl from the pyruvate that originates in the glycogenesis pathway (Lerm et al., 2011; Mink et al., 2015). Cit^+^ LAB strains typically produce more D-lactate, acetate, diacetyl and acetoin from pyruvate (Pretorius et al., 2019). The content of diacetyl found in wine is therefore dependent on the LAB species used as a starter for MLF (Lonvaud-Funel, 1999; Bartowsky and Henschke, 2004).

### Glycosidase Activity

**FIGURE 2:**
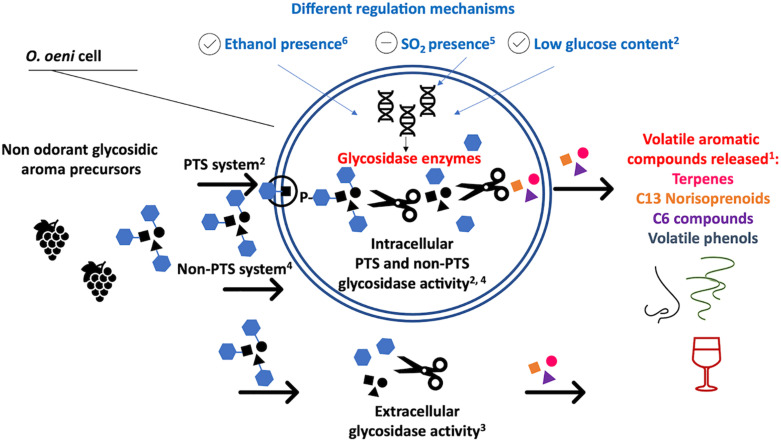
Reported glycosidase activity in *O. oeni*. ^1^Baumes, 2009; ^2^Capaldo, 2012; ^3^Grimaldi et al., 2000; ^4^Michlmayr et al., 2010a,b; ^5^Spano et al., 2005; ^6^Grimaldi et al., 2000.

Odorless, sugar-bound aromas represent a reservoir of wine aroma that can be released by cleaving the bonds between the glycosidic molecule and the volatile, aromatic aglycone (Figure 2), such as terpenes, C13 norisoprenoids, volatile phenols, C6 compounds and others (Baumes, 2009). A group of enzymes called glycosidases are responsible for the enzymatic hydrolysis of these compounds. The type of aroma precursor determines which specific glycosidase is needed to break the bonds. The aromatic volatiles can be conjugated to glucose (β-D-glucopyranoside) or to a disaccharide. In the latter case, the inner molecule is a glucose unit and the outer one is a second sugar unit (e.g., α-L-arabinofuranose, α-L-rhamnopyranose, β-D-xylopyranose, or β-D-apiofuranose) (Williams et al., 1982; Gunata et al., 1988). When the aromatic compound is linked to a glucose, a β-glucosidase is required to hydrolyze the bond. When the aromatic compound is linked to a disaccharide two enzymes are needed to break the bond: an exo-glycosidase e.g., α-L-arabinofuranosidase, α-L-rhamnopyranosidase, β-D-xylopyranosidase, or β-D-apiofuranosidase, cleaves the second sugar unit and subsequently a β-glucosidase removes the remaining glucose (Gunata et al., 1988).

Glucosidases can originate from different sources, including grapes (Aryan et al., 1987), yeasts (Swiegers et al., 2005) and bacteria (Bartowsky and Borneman, 2011; Capaldo, 2012; Cappello et al., 2017). The addition of enzymatic preparations with hydrolytic activity to musts and wines can increase the content of free aromatic compounds such as terpenes and norisoprenoids (Pretorius and Rensburg, 2000; Codresi et al., 2012). However, purified enzymes can be expensive and may have low specificity and efficiency (Tavernini et al., 2020). To overcome these issues, polymeric chitosan beads have been successfully trialed as matrix for the immobilization of β-D-glucosidases and α-L-arabinofuranosidases sourced from a commercial, *Aspergillus niger*-derived preparation and previously organized in a cross-linked matrix (Tavernini et al., 2020). There is also a high potential for the use of glycosidases from bacteria, especially LAB, because these enzymes could perform better under stressful conditions such as high concentrations of alcohol and acidity (Bartowsky and Borneman, 2011).

Several authors have shown that LAB are able to enzymatically hydrolyze glycosides and that this activity is widespread amongst different *O. oeni* strains (Grimaldi et al., 2000, 2005a; Barbagallo et al., 2004; D’Incecco et al., 2004; Spano et al., 2005; Hernandez-Orte et al., 2009; Fia et al., 2018) and other LAB genera (Grimaldi et al., 2005b; Spano et al., 2005; Hernandez-Orte et al., 2009). *Lactiplantibacillus* strains show a varied array of enzymes that can contribute to wine aroma, and particularly, a great ability to release volatile aromas from glycosidic precursors (Lerm et al., 2011; Iorizzo et al., 2016).

Although considerable efforts have been directed toward understanding the genetics and the expression mechanisms, the glycosidase activity of LAB is not yet clear. The carbohydrate phosphotransferase system (PTS) seems to play a key role in *O. oeni*, by phosphorylating and transporting the glycosides into the cell (Figure 2) through a membrane-associated enzymatic system (Capaldo, 2012). The activated glycosides can then be hydrolyzed by intracellular phosphoglycosidases (Capaldo, 2012). Some phosphoglycosidases have been characterized and their hydrolytic activity against *p-*nitrophenol-β-D-glucopyranoside-6-phosphate confirmed (Capaldo, 2012). Intracellular non-PTS glycosidase activity has been reported by a number of authors (Figure 2) and some of these glucosidases have also been characterized (Michlmayr et al., 2010a,b; Capaldo, 2012). However, other authors reported extra-cellular and parietal glycosidase activity, suggesting that the glycosidase activity in *O. oeni* is the consequence of a complex enzymatic system, involving many different genes, enzymes and regulation mechanisms (Grimaldi et al., 2000; Barbagallo et al., 2004). Little is known regarding the molecular mechanisms involved in the glycosidase activity of other LAB, such as *Lactiplantibacillus* and *Pediococcus*. It is known that abiotic stresses have a key role in upregulating the expression of these enzymes, although data are controversial. Low levels of glucose and the presence of ethanol seem to lead to an enhancement of the glycosidase activity, while low pH values and sulfite content seem to repress the activity (D’Incecco et al., 2004; Grimaldi et al., 2005a,b; Spano et al., 2005; Capaldo, 2012). See Figure 2 for an overview of the glycosidase activity of LAB in wine.

Finally, it is important to note that glycosidase activities in wine can also have a negative effect on the final product. Similar to yeast, the bacterial starters, with their ability to hydrolyse the glycoconjugate compounds, can also heavily affect the evolution of volatile phenols associated with smoke taint, influencing the intensity of smoke taint-associated aromas (Kennison et al., 2008; Jiranek, 2011). The aroma compounds associated with the smoky and earthy notes that characterize smoke tainted wines, such as guaiacol, 4-methylguaiacol, 4-ethylguaiacol and 4-ethylphenol, typically peak in finished wine (388 μg/L for guaiacol and 93 μg/L 4-for methylguaiacol) (Kennison et al., 2007). This can be explained by the presence of conjugated precursor compounds, particularly disaccharide conjugates, which can be liberated via enzymatic hydrolysis (Kennison et al., 2008; Hayasaka et al., 2010; Dungey et al., 2011). For the impact that LAB glycosidase activity can have on wine color see the dedicated paragraph: “how can LAB impact the wine colour?”.

### Release of Volatile Thiols From Precursor Compounds

Volatile thiols, such as 3-sulfanylhexan-1-ol and 3-sulfanylhexyl acetate, first identified in Sauvignon Blanc, are important aromatic compounds that contribute varietal aromas like grapefruit and passionfruit (Takase et al., 2018). Their non-odorant precursors, such as glutathione S-conjugate, cysteinyl-glycine S-conjugate and cysteine S-conjugate, are found in grapes and are enzymatically released by yeast during AF (Thibon et al., 2008). A 2019 study has shown that *L. plantarum* is able to enzymatically convert the cysteine S-conjugates and cysteinyl-glycine S-conjugates, with a noticeable preference for the latter compound under experimental conditions (Takase et al., 2018). The enzymes responsible for this transformation and the underlying regulatory mechanisms have not yet been characterized. However, this represents an opportunity to improve the varietal aroma of wines, and further research is warranted to provide a better understanding of this metabolic pathway.

### Esterase Activity

Esters are secondary or tertiary aroma compounds that contribute significantly to wine aroma. In wine, esters can either be formed in a process called esterification, or broken down via ester hydrolysis (Waterhouse et al., 2016). They can be formed during the primary or secondary fermentation by yeast or bacteria, and their concentration and composition is slowly changed during wine aging (Liu, 2002; Sumby et al., 2010, 2013; Antalick et al., 2012). The contribution of LAB to the ester composition of wine has been highlighted with several wine volatile profiling studies conducted after MLF (Ugliano and Moio, 2005; Antalick et al., 2012; Costello et al., 2012; Sumby et al., 2012; Lytra et al., 2020). In wine, LAB can both increase and decrease the content of esters (Sumby et al., 2013). The degree of LAB contribution to the ester profile of wine is strain-specific (Sumby et al., 2013; Gammacurta et al., 2018) and MLF inoculation strategy can affect the quantity and quality of esters released by the bacteria (Lasik-Kurdyś et al., 2018). It seems that the use of a coinoculation technique increases the release of ethyl esters, particularly ethyl lactate, diethyl succinate and ethyl acetate, thus (depending on their concentration) enriching the wine with floral and fruity notes (Lasik-Kurdyś et al., 2018).

### Activities That Can Affect the Content of Volatile Phenols in Wine

Ethyl phenols are crucial aromatic compounds associated with unpleasant odors in wine such as horse sweat, leather and stable. Ethyl phenols are produced by some yeasts within the genus *Brettanomyces*/*Dekkera* from hydroxycinnamic acids (HCAs) precursors (Chatonnet et al., 1992). *Brettanomyces* yeasts can decarboxylase the free HCAs, converting them into vinylphenols first, via an hydroxycinnamate decarboxylase enzyme (HCDC), and then into the unpleasant ethyl phenol, via a vinylphenol reductase enzyme (VPhR) (Chatonnet et al., 1992). HCAs can be found in the must as free acids but, most commonly, as their tartrate esters, which are hydrolyzed slowly throughout the winemaking process (Waterhouse et al., 2016; Lima et al., 2018). This leads to small amounts of ethyl phenol precursors being continuously released in wine and made available to *Brettanomyces* metabolism.

It has been reported that the use of *O. oeni* strains with cinnamoyl esterase activity can lead to higher amounts of 4-ethylphenol and 4-ethylguaiacol in Pinot Noir after MLF, compared to wine that did not undergo MLF, or underwent MLF with bacterial starters with no cinnamoyl esterase activity (Chescheir et al., 2015). Cinnamoyl esterase activity is strain-specific in *O. oeni* and may be constitutively expressed (Chescheir et al., 2015; Collombel et al., 2019). Thus, to avoid the faults that arise from *Brettanomyces* spoilage, it can be beneficial to use bacterial starters with low cinnamoyl esterase activity (Chescheir et al., 2015).

LAB have also been reported to be directly responsible for the production of 4-vinylphenol (Silva et al., 2011). In *L. plantarum*, *Secundilactobacillus collinoides* (formerly known as *Lactobacillus collinoides)* and *Pediococcus pentosaceus*, this activity is enhanced by the presence of hydroxycinnamic acids, especially caffeic acid, in the growth medium (Silva et al., 2011). Conversely, a tannin content of 1 g/L (the average range for red wines is 1–4 g/L) can inhibit the release of volatile phenols in wine by *L. plantarum* (Silva et al., 2011). However, the literature regarding this matter is still controversial. It has also been suggested that the addition to the must of cinnamoyl esterase enzymes can help reduce the formation of ethyl phenols (Morata et al., 2013). The intermediate vinylphenols are also able to react with anthocyanins, producing very stable vinylphenolic pyranoanthocyanins (Rentzsch et al., 2007). When vinylphenols are present in their bound form, they can help reduce the number of precursors for the formation of ethyl phenols and also stabilize the color of wine (Morata et al., 2013). In support of this, wines fermented with *Saccharomyces* yeast with an increased hydroxycinnamate decarboxylase activity (HCDC+) have an increased content of vinylphenolic pyranoanthocyanins and a reduced content of ethyl phenols (Morata et al., 2013). The addition to the must of cinnamoyl esterase enzymes, to help release more quickly the free HCAs paired with the use of HCDC+ *Saccharomyces cerevisiae* strains, could be a way to reduce the formation of ethylphenols (Morata et al., 2013). Similarly, the use of LAB with enhanced cinnamoyl esterase activity could be beneficial for the quality of wine, helping to prevent the formation of unwanted off flavors and favoring the development of stable color compounds.

Furthermore, the levels of volatile phenols in the finished wine seem to depend more on differences between *Brettanomyces* strains rather than on the cinnamoyl esterase activity of the LAB employed for MLF (Madsen et al., 2017). Two different *O. oeni* strains, with and without cinnamoyl esterase activity, were tested on Cabernet Sauvignon and, although there were differences in the degradation rate of tartaric esters of HCAs into free HCAs, this did not affect the final content of volatile phenols (Madsen et al., 2017).

Further studies are needed to characterize the cinnamoyl esterase activity in LAB. Understanding the basis for the differential cinnamoyl esterase activity of LAB could enable the winemaker to make appropriate decisions in regards the choice of bacteria to employ.

### Acetaldehyde Metabolism

Acetaldehyde is an important compound in wine, affecting aroma, color, stability and microbiological properties (Liu and Pilone, 2000). Wine typically contains 20-100 mg/L of acetaldehyde, and, at low concentrations, it can enhance the fruity character of wine (Waterhouse et al., 2016). However, at higher concentrations it is associated with an unpleasant rotten apple aroma (Waterhouse et al., 2016). Acetaldehyde is an intermediate compound produced by yeast during AF and also plays a vital role in the stabilization of wine color (see next section “how can LAB impact the wine color?”) (Waterhouse et al., 2016; Forino et al., 2020). During AF acetaldehyde is readily reduced to ethanol, thus at this stage, its content in wine is typically low (25–40 mg/L) depending on yeast strain and the concentration of SO_2_ in the must (Waterhouse et al., 2016). However, in later stages, the oxidation of alcohol can lead to an increase in acetaldehyde content (Waterhouse et al., 2016).

Excessive accumulation of acetaldehyde is not desirable because it strongly binds to sulfur dioxide making it less active (see section “reducing the SO_2_ required in winemaking”) and requiring the winemaker to add additional SO_2_ to protect the wine (Wells and Osborne, 2011). Additionally, as acetaldehyde has potential toxic and carcinogenic effects, high amounts are undesirable in beverages for human consumption (Lachenmeier and Sohnius, 2008).

Acetaldehyde can exert a stimulating or inhibiting effect on LAB growth in wine (Liu and Pilone, 2000). Both heterofermentative and homofermentative LAB strains can degrade free and SO_2_-bound acetaldehyde into small amounts of ethanol and acetic acid (Osborne et al., 2000). However, this could also lead to higher VA in wine (Waterhouse et al., 2016). The efficiency of this activity seems to be strain-specific (Mira and de Orduña, 2010). The ability of probiotics, including LAB, to break down acetaldehyde, is therefore of interest to the wine industry and could be used to reduce acetaldehyde levels in wine. Indeed, a patent describing “a composition of probiotics including LAB, able to degrade alcohol and acetaldehyde,” to help prevent and treat alcohol-related diseases, has been filed (Chung, 2018).

## How Can Lab Impact the Wine Color?

Wine color is an important sensory attribute (Figure 1) that is relevant to red wines, largely related to the grape variety and the vintage, and to a minor extent to the winemaking practices (González-Neves et al., 2013). Wine color can also be affected by the activity of yeast and LAB (Morata et al., 2005; Benito et al., 2011; Burns and Osborne, 2013, 2015; Devi and Ka, 2019; Devi et al., 2019). Color loss is common in wines that have undergone MLF (Abrahamse and Bartowsky, 2012; Burns and Osborne, 2013, 2015). Independently from pH, wines post-MLF have lower levels of polymeric pigments, lower Visitin A and B content and a higher content of monomeric anthocyanins than their respective controls that did not undergo MLF (Burns and Osborne, 2013). An explanation for the color loss in wine post-MLF is the LAB metabolism of acetaldehyde (see acetaldehyde metabolism above) (Burns and Osborne, 2015). Acetaldehyde is crucial for the formation and stabilization of wine color because it mediates the formation of stable ethylene-linked pigments, which are more stable than their respective monomeric anthocyanins and show better colorimetric properties (Forino et al., 2020). Furthermore, pyruvic acid and acetaldehyde can react with pigments such as malvidin-3-glucoside, generating the relatively stable pyranoanthocyanins Visitin A and Visitin B (Waterhouse et al., 2016).

Recent studies have shown that *O. oeni* and *L. plantarum* strains can also absorb anthocyanin glucosides such as delphinidin-3-glucoside, malvidin-3-glucoside and peonidin-3-glucoside through the cell wall (Devi et al., 2019). These LAB can produce β-glycosidase enzymes that cleave the anthocyanin glucoside glycosidic bonds and can further degrade the aglycons into phloroglucinol aldehyde and corresponding phenolic acids (Devi et al., 2019). The absorption rate of anthocyanin glucosides, the β-glycosidase activity and the degradation rate of anthocyanins are dependent on the species and strains of LAB (Devi et al., 2019). Different inoculation regimes, such as coinoculation or sequential inoculation of yeast and bacteria, can determine different wine color outcomes, seemingly due to microbial interactions, rather than to absorption mechanisms (Devi and Ka, 2019). A higher color loss occurs when MLF is performed with sequential inoculation regimes, rather than with coinoculation regimes (Abrahamse and Bartowsky, 2012).

Little has been reported regarding the long-term effects of MLF on wine color. A 2016 study reported that, nine months after MLF completion, up to 9% color intensity loss and lower acylated and non-acylated anthocyanins levels were detected in wines (Izquierdo-Cañas et al., 2016). Interestingly an increase of the pyranoanthocyanin concentration was also observed (Izquierdo-Cañas et al., 2016).

Further research is warranted to understand the specific yeast-bacteria interactions during the secondary fermentation, and how they subsequently affect wine color. Considering the multitude of factors involved, understanding the basis of wine color is not simple and far from fully achieved. However, this knowledge would greatly benefit the industry by enabling the winemaker to plan MLF in order to obtain the desired color outcome even in wines naturally low in pigments.

## Possible Contributions of Lab to Winemaking

### Reducing the Need for Bentonite

Residual grape proteins can represent a problem for the quality of wine. Thaumatin-like proteins, chitinases and, to a lesser extent, β-glucanases, are the main classes of proteins responsible for wine protein instability (Van Sluyter et al., 2015; Cosme et al., 2020). Protein aggregation, particularly during wine storage, can lead to the formation of an unwanted sediment or haze (Figure 1) once the wine is bottled (Ferreira et al., 2001). Haze formation in white wines is mainly an esthetic issue, but it can economically depreciate the final product (Van Sluyter et al., 2015).

To alleviate this problem, the main treatment currently applied during winemaking is the addition of bentonite. However, this is a costly treatment and it involves a loss of wine volume, wine aroma and assimilable nitrogen resources (Muhlack et al., 2006). It has been shown that proteolytic and peptidolytic activities are common amongst LAB (Savijoki et al., 2006; Moslehishad et al., 2013; Atanasova et al., 2014; García-Cano et al., 2019). LAB proteolytic activities could be exploited in winemaking and have the potential to replace or reduce the use of fining agents such as bentonite. However, more studies are needed as most of the literature refers to work done in the dairy industry, at pH values of 6.5 and with considerably different substrates (Moslehishad et al., 2013; Atanasova et al., 2014; García-Cano et al., 2019).

### Aiding Clarification and Filtration of Juice

Grape juice is naturally rich in polysaccharides, such as pectin, cellulose, hemicellulose, and other substances, originating mainly from grape cell walls and the middle lamellae. In particular, the high content of pectins leads to the formation of a colloid structure, which can make processing of juice difficult (Sandri et al., 2011). The addition of fungal pectinase enzymes can help with the clarification and filtration of the juice, increasing must yield, and favoring the extraction of polyphenols, pigments and aromas (Merín et al., 2011). Some yeast strains are capable of degrading polysaccharides (Merín et al., 2014; Belda et al., 2016). Similarly, LAB may also possess pectinolytic activity, however, little research has been done in this field (Ruiz Rodríguez et al., 2019).

### Reducing the SO_2_ Required in Winemaking

Due to its antimicrobial and antioxidant properties, SO_2_ is one of the most common agents added to wine (Lisanti et al., 2019). Despite the benefits that come from SO_2_, the sensitivity of some consumers to this compound, have raised concerns regarding its safety in the food and beverage industries (Benito, 2019). Thus, the addition of potassium metabisulfite to wine is strictly regulated, with maximum limits varying depending on local regulations. As a general guide, the international code of enological practices recommends a residual limit of 150 mg/L total sulfur dioxide for red wines and 200 mg/L for white and rosé wines with up to 4 g/L of reducing substances^3^.

As mentioned already, acetaldehyde strongly binds free SO_2_, making it less effective in its antiseptic, antioxidant and antioxidasic roles (Ribéreau-Gayon et al., 2006b). Thus, reducing the content of acetaldehyde in wine could help minimize the amount of SO_2_ required in winemaking (Figure 1) and, at the same time, could improve the aroma of wine (Lisanti et al., 2019). The use of LAB starters that are able to degrade SO_2_-bound acetaldehyde (see acetaldehyde metabolism above) could represent a winemaking strategy to minimize SO_2_ additions to wine by converting the bound SO_2_ into the more effective free SO_2_. Importantly, acetaldehyde also plays a crucial role in the development of wine color. Thus, its content in wine has to be optimized to determine the balance between optimizing color, flavors and aromas, and health associated risks.

## The Impact of Lab on the Wholesomeness of the Wine

**FIGURE 3:**
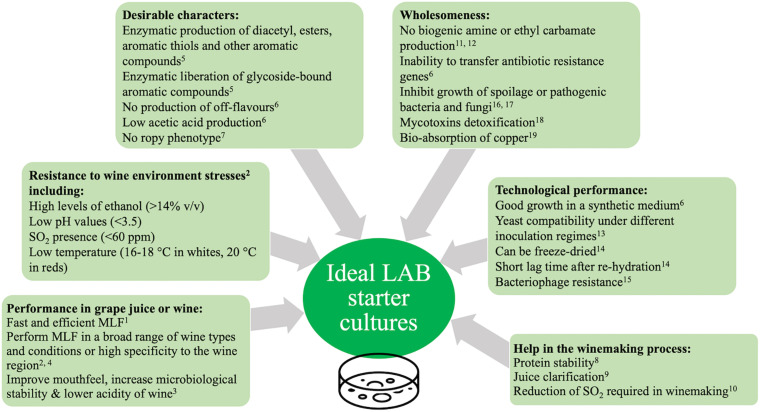
Guidelines for the selection of MLF starters for use in winemaking. Adapted from Henick-Kling, 1995 and Torriani et al., 2011. ^1^Henick-Kling, 1995; ^2^Costello et al., 2015; ^3^Lonvaud-Funel, 1999; ^4^Bartowsky et al., 2015; ^5^Matthews et al., 2004; ^6^Torriani et al., 2011; ^7^Wade et al., 2018; ^8^García-Cano et al., 2019; ^9^Ruiz Rodríguez et al., 2019; ^10^Lisanti et al., 2019; ^11^Callejón et al., 2014; ^12^Araque et al., 2009; ^13^Bartle et al., 2019; ^14^Fonseca et al., 2015; ^15^Mozzi et al., 2015; ^16^Szutowska, 2020; ^17^Bianchini, 2015; ^18^Muhialdin and Saari, 2020; ^19^Harald, 2020.

LAB can positively and negatively affect the impact that wine may have on human health (Figure 3). LAB can produce numerous metabolites, including organic acids (mainly lactic and acetic), phenyllactic acid, diacetyl, cyclic dipeptides, and bacteriocins that can inhibit the growth of spoilage or pathogenic bacteria (Corsetti et al., 2015; Szutowska, 2020). They have also been reported to have antifungal activity, although the mechanisms of action are not clear (Bianchini, 2015). LAB can exert a high detoxifying action toward mycotoxins in various foods including wine (Muhialdin and Saari, 2020). This is likely to be due to the proteolytic activity of LAB, however, other detoxification mechanisms have been suggested, including the binding of mycotoxins to LAB metabolites such as acids, phenolic compounds and small bioactive peptides, and the absorption of mycotoxins by the bacterial cell walls (Muhialdin and Saari, 2020). Recent studies show that wines that underwent MLF have higher contents of melatonin and other tryptophan related compounds, which are associated with several human health benefits (Fracassetti et al., 2020). LAB could also have a role in the bio-absorption of copper, which, at high concentrations, is associated with health risks and negative effects in wine too (Harald, 2020).

### Biogenic Amines

Biogenic amines (BA) are potentially hazardous compounds that are commonly found in wine (Figure 1) and other fermented foods (Benito, 2019). Although BA can be produced in the grapes (Del Prete et al., 2009) and by yeast (Tristezza et al., 2013), their presence in wine is primarily due to LAB metabolism (Costantini et al., 2019). The main BAs produced during MLF are histamine, putrescine and tyramine (Landete et al., 2007).

These BAs are formed through the activity of the LAB enzymes, histidine decarboxylase (*hdc*), ornithine decarboxylase (*odc*), and tyrosine decarboxylase (*tdc*), which decarboxylate the precursor compounds histidine, ornithine and tyramine, with both the enzymatic activity and presence of the genes encoding these proteins being strain dependent (Coton et al., 2010; Costantini et al., 2019). In addition to this, several LAB genera can produce putrescine via the agmatine deiminase, from its precursor agmatine (*agdi*) (Lucas et al., 2007).

Some LAB can degrade BA in culture media, and to different degrees in wine, through the action of multicopper oxidases or amine oxidases (García-Ruiz et al., 2011; Callejón et al., 2014). Many species, including *Levilactobacillus brevis* (formerly *Lactobacillus brevis*), *L. delbrueckii*, *L. hilgardii*, *L. paracasei*, *Lactiplantibacillus pentosus* (formerly *Lactobacillus pentosus*), *L. plantarum*, *P. parvulus*, and *P. pentosaceus* have been biochemically tested and showed degrading activity against BA (García-Ruiz et al., 2011; Callejón et al., 2014). Seemingly this property of LAB is strain dependent for most species tested, except in the case of *L. plantarum*, in which a high level of activity was widespread amongst all strains (Callejón et al., 2014). Similar results were obtained in a previous study which aimed to investigate the BA degrading activity of two *L. plantarum* strains (NDT 09 and NDT16) (Capozzi et al., 2012). Interestingly, the degrading activity of *L. plantarum* NDT09 and *L. plantarum* NDT16 toward putrescine and tyramine, in synthetic media, was higher when the two strains were used in conjunction (Capozzi et al., 2012). Recently, a *L. plantarum* with good MLF and stress tolerance properties, that is also able to decrease the histamine, tyramine and cadaverine content by over 57%, has been obtained, through successive screenings (Sun et al., 2020).

Currently, the options available to control the BA content in wine are the reduction of precursor compounds, the inhibition of spoilage bacteria growth and the use of selected non biogenic amine-producer starters (Callejón et al., 2014). Given the recent findings on the ability of LAB, particularly *L. plantarum* strains, to degrade BA, the use of MLF starters that are able to reduce BA in wine would represent an innovative and alternative option for the winemaker.

### Ethyl Carbamate

Ethyl carbamate (Figure 1) is a carcinogenic compound found in many fermented foods and beverages (Benito, 2019). The reaction of ethanol with N-carbamyl compounds, such as urea (produced by yeast) and citrulline (produced by some LAB), is responsible for the main formation of ethyl carbamate in wine (Liu et al., 1994).

Some species of LAB can degrade L-arginine, through the arginine deaminase pathway, producing ATP, CO_2_ and ammonium as final products (Araque et al., 2009). The intermediate steps in this pathway yield potential ethyl carbamate precursors like citrulline and carbamyl phosphate (Liu et al., 1994; Araque et al., 2009). The genes *arcA*, *arcB*, and *arcC*, which encode for the enzymes arginine deiminase, ornithine transcarbamylase and carbamate kinase, respectively, have been characterized in many genera (Araque et al., 2009). The activity of these genes, and the production of ethyl carbamate precursors, has been reported for all wine-related genera (Liu et al., 1994, 1995; Araque et al., 2009).

The presence of the *arc* genes is generally a good predictor of the capability of LAB to degrading arginine (Liu et al., 1995; Araque et al., 2009); however, it is not indicative of the expression of the genes under particular circumstances. Both genotypic and phenotypic screenings are recommended to correctly characterize the capability of LAB to produce ethyl carbamate precursors in wine. On the other hand, LAB can produce urea-degrading enzymes that can selectively hydrolyze urea, contributing to reduction of ethyl carbamate in wine (Fang et al., 2016).

## Improvement of Lab and Technological Advances

### Strain Selection

The selection of new LAB starters requires the assessment of many properties related to the new strain including resistance to biotic and abiotic stresses, technological performances and safety aspects (Henick-Kling, 1995; Torriani et al., 2011; Costello et al., 2015). See Figure 3 for a summary of the properties of interest. Traditionally a phenotypic approach has been used for the purpose of selecting new starters (Torriani et al., 2011). In recent years, phenotypic and genotypic approaches are used in tandem to rapidly characterise new potential starter candidates (Torriani et al., 2011).

### Microbial Integrity: Phage Resistance

One of the major issues that can arise during fermentation processes is the contamination of the LAB starter cultures with bacteriophages (Mozzi et al., 2015). Most of the knowledge regarding phage infections in LAB comes from the dairy industry, with *Lactococcus lactis* and *Streptococcus thermophilus* being the most susceptible and studied species (Mozzi et al., 2015). However, since *O. oeni*, *Lacticaseibacillus casei* (formerly known as *Lactobacillus casei), L. delbrueckii* and other species have become more prominent in the food industry, more research has been directed toward investigating their phages, disclosing multiple phage infections within these genera (Mozzi et al., 2015). In a 2017 study, 15 *O. oeni* phages have been studied, containing more than one type of integrase genes each and varying in genome size (Costantini et al., 2017). In the same study, it was shown that the presence of ethanol and low pH values inhibited the phages from attacking the cells, suggesting that inoculation time of the bacterial starters could play a significant role in preventing bacteriophage infections (Costantini et al., 2017). The first sequence of an *O. oeni* phage, E33PA, has been published in 2018 by Jaomanjaka and co-workers (Jaomanjaka et al., 2018). Interestingly, although most of phage isolated from the *Oenococcus* genus are temperate, E33PA was found to have a predominately lytic lifestyle (Jaomanjaka et al., 2018).

The adaptive immune system of bacteria is a potential mechanism for defense against phage, which has been highlighted by recent studies (Barrangou and Marraffini, 2014). For example, it is now known that Clustered Regularly Interspaced Short Palindromic Repeats (CRISPR) and CRISPR-associated genes, are essential elements in phage defense (Barrangou and Marraffini, 2014). CRISPR-Cas systems can confer phage resistance to the bacterial cell through three steps: during the adaptation, foreign genomic sequences can be integrated in the CRISPR arrays as new spacers, bordered by the palindromic repeats. During the expression the CRISPR locus is transcribed and processed, generating multiple copies of small CRISPR RNAs, able to direct the Cas proteins to foreign, complimentary DNA. Lastly, during the interference, foreign nucleic acid is targeted and cleaved (Crawley et al., 2018).

The CRISPR-mediated immunity mechanism causes phages to mutate at high rates, to maintain their ability to infect the hosts; furthermore, it’s likely that phages and CRISPR systems are continuously co-evolving (Sorek et al., 2008). Interestingly, there is a rich variety of type II CRISPR-Cas systems within LAB (Sun et al., 2015); in a 2015 study comprising 213 different LAB genera, type II systems were found in 36% of them, representing a wealth of tools for LAB genetic engineering (Sun et al., 2015).

Conversely, CRISPR-Cas systems are either uncommon or absent in *O. oeni*. We searched six different *O. oeni* whole genome sequences from NCBI on the CRISPR finder database (version 4.2.20) (Couvin et al., 2018) showing that there are no CRISPR arrays or Cas clusters.

However, two closely related *Oenococcus* species contained both Cas clusters and CRISPR arrays at a high-quality score (quality score of 4). These were *O. kitaharae* DSM 17330 (CAS clusters = 1, CRISPR arrays = 1) and *O. sicerae* UCMA 15228 (CAS clusters = 2, CRISPR arrays = 2).

This inconsistent distribution of CRISPR-Cas immune systems within and between bacterial species is not uncommon and it may be explained by the fact that bacterial cells that have lost the CRISPR-Cas system from their genome avoid damage caused by autoimmune targeting (Rollie et al., 2020). Additionally, the efficacy of CRISPR-Cas is dependent on viral mutation rate and the frequency of spacer incorporation. *O. oeni* has a slow growth rate (∼7 days to reach OD_600_ of 1.0) and may not be able to mount a response fast enough or acquire the spacers quickly enough for the system to be effective (Sorek et al., 2008).

### Strategies to Harness LAB Enzymatic Activity

Directed evolution of microorganisms is a widely used technique for improving desired properties of bacterial strains (Bachmann et al., 2017). It is based on the concept that, during evolution, the environment selects the fittest variants. Growth limiting conditions, such as scarcity of nutrients, and environmental stresses induce a stress response and can lead to the production of genetic mutations (Rosenberg and Hastings, 2003). To generate an array of genetic and phenotypic variants treatments of UV and ethylmethanesulfonate (EMS) can also be applied to the culture (Dragosits and Mattanovich, 2013; Li et al., 2015).

As part of a broader study involving metabolic networks, a strain of *L. plantarum* has been successfully adapted, through serial dilutions, to grow well in a medium containing glycerol as main carbon source (Teusink et al., 2009). Directed evolution has been used to successfully generate acid-resistant mutants of *L. casei* and *Leuconostoc mesenteroides* (Zhang et al., 2012; Ju et al., 2016). An ethanol tolerant *O. oeni* strain, A90, has been generated after exposure to increasing ethanol concentrations, in approximately 330 generations (Betteridge et al., 2018). This strain was further evolved to withstand the multiple stresses typical of wine environment, obtaining after approximately 350 generations, an alcohol tolerant, acid tolerant (3.35 pH) and SO_2_ tolerant (26 mg/L) strain (Jiang et al., 2018).

Directed evolution, as a strategy to harness LAB enzymatic activity, offers several advantages (Bennett, 2002). It does not involve the use of recombinant technology, posing less problems in the public acceptance of the products. It does not require a specific knowledge of the underlying genetics behind the specific phenotypic traits that are object of interest. Finally, whereas bioengineering approaches often target one gene at the time, directed evolution can result in multiple beneficial mutations, broadening the possible outcomes (Bloom and Arnold, 2009). However, the specific application of directed evolution to the LAB species associated with wine, is still a relatively new but promising technique. By using this approach, it could be possible to produce LAB strains that exhibit a higher desired enzymatic activity and/or are more tolerant to the harsh wine environment.

Another way to enhance the enzymatic activity of bacteria is the immobilization of the organisms onto supports such as alginate beads or apple pieces, corn cobs, delignified cellulosic material, grape skins and grape stems (Genisheva et al., 2013; Bleve et al., 2016; Simó et al., 2017; Nikolaou et al., 2020). Immobilized bacteria can perform MLF twice as fast, compared to free cells, and are also more resistant to ethanol content, SO_2_ content and elevated temperatures (Genisheva et al., 2013).

Currently, it may not be practical to apply this methodology in a traditional winemaking system, considering the large amounts of required supporting material (up to 30 g/L). Nonetheless this is a promising technique, with a great potential to improve MLF in wine. Furthermore, it would be interesting to know if secondary enzymatic activities of LAB would also be affected by the use of immobilized cells.

### Genome Editing of LAB

In addition to strain selection and non-GMO strategies to harness enzymatic activity of LAB, genome editing is a promising strategy to develop high performing bacterial starters. Transformation with plasmids, transduction and conjugative transposons systems are the main recombinant methods used to genetically manipulate LAB (Sumby et al., 2014). The transformation of *Lactiplantibacillus* and *Lacticaseibacillus* strains has been successfully performed by many authors (Spath et al., 2012; Xin et al., 2018). However, the rate of success of this method depends on the plasmid vector, on the bacterial strain and the possible presence of a enzymatic restriction system in the host cell (Teresa Alegre et al., 2004). Currently, not many expression vectors are available for *O. oeni* (Sumby et al., 2014). A plasmid, pGID052, was developed in 2004 and successfully mobilized from *L. lactis* to *O. oeni* (Beltramo et al., 2004). The same plasmid, pGID052, encoding a truncated form of the ClpL2 protein was later introduced in *O. oeni* ATCC BAA-1163, trialing an optimized electroporation method (Assad-García et al., 2008). Although promising, there has been no follow up research to these findings, perhaps due to the low copy level of this plasmid (Sumby et al., 2014).

The most promising technology is currently based on CRISPR-Cas systems. This strategy exploits the functions of CRISPR and CRISPR-associated genes (Doudna and Charpentier, 2014; van Pijkeren and Barrangou, 2017). Desired edits can be obtained with different methods, e.g., utilizing a plasmid-encoded recombineering sequence or an oligonucleotide sequence with an inducible DNA recombinase (Leenay et al., 2019). The efficiency of the selected method varies dramatically across different strains of *Lactoplantibacillus plantarum* (Leenay et al., 2019).

### Bacterial Starter Implementation

Freeze drying is the method of choice for long term storage of bacteria and continuous research has been done toward the optimization of the process (Fonseca et al., 2015, 2019; Polo et al., 2017). Freeze dried bacterial starters have a good rate of cell viability over long periods and are easy to use, store and transport (Fonseca et al., 2015). To protect the integrity of the cells membranes and the structural conformation of proteins and DNA during the dehydration process, lyoprotectans such as trehalose, sucrose and monosodium glutamate, are typically added (Wang et al., 2019). More recently, soluble extracellular polymeric substances (sEPS) from *O. oeni* have been successfully tested as lyoprotectans. Notably, a mixture of 5% sEPS, 15% sucrose, 15% trehalose, and 0.5% MSG, increased the cell viability to 92.83% (Wang et al., 2019). The positive effects of ethanol acclimatization on cell survival rate of lyophilized *O. oeni* have also been reported (Yang et al., 2020). Due to the length and costs associated with this process, other techniques have been investigated, including vacuum drying, spray drying, drum drying, fluidized bed drying and air drying (Santivarangkna et al., 2007). Currently these alternative drying processes do not perform as well as freeze drying, especially regarding the cell viability and the lag time required to restore the metabolic activity (Santivarangkna et al., 2007). However, they could represent a cheaper, fast and efficient way to preserve commercial bacterial starters. Promising results have also been obtained by freeze drying bacterial cultures previously immobilized onto natural supports (Nikolaou et al., 2020).

## Limitations and Future Prospects

Currently the main enological role of LAB is performing MLF. Thus, the use of LAB, with the intention of increasing wine aroma and facilitating the winemaking process, is an unexplored and promising field. In this new broader role, LAB could replace, or partially substitute, the use of purified enzymatic preparations. Furthermore, using whole cells, rather than purified enzymes, provides a series of advantages (De Carvalho and da Fonseca, 2007).

Firstly, whole cells are a natural environment for enzymes, preventing loss of activity in non-conventional media. Secondly, they are able to efficiently regenerate co-factors. However, more studies are needed in order to improve the knowledge of LAB’s secondary enzymatic activities and their potential usability in the production of wine.

To benefit from the LAB’s secondary metabolism a system to control and enhance the expression of these activities is needed.

Currently, disregarding studies on recombinant technology, little research has been done on this point (Renault, 2002). Directed evolution has been successfully used to produce highly performing LAB strains in the dairy industry, but seldomly in the wine industry (Betteridge et al., 2015). However, without resorting to genetic engineering techniques, no other methods are currently available to enhance a chosen secondary enzymatic activity in a LAB strain.

Overall, the benefits of harnessing the enzymatic activities of LAB will increase the options open to winemakers to sculpt the wine of their choice. The industry could benefit greatly from the advent of these extra tools.

## Author Contributions

CV and KS prepared the first draft. All authors reviewed and finalized the manuscript. All authors contributed to the development of the outline and scope of the review.

## Conflict of Interest

EB was employed by Lallemand Australia. The remaining authors declare that the research was conducted in the absence of any commercial or financial relationships that could be construed as a potential conflict of interest.

## Footnotes

1 www.lallemandwine.com

2 www.chr-hansen.com

3 http://www.oiv.int
